# Laparoscopic transgastric necrosectomy in treatment of walled-off pancreatic necrosis with sinistral portal hypertension

**DOI:** 10.1186/s12893-021-01361-6

**Published:** 2021-10-10

**Authors:** Feng Cao, Ang Li, Xiaohui Wang, Chongchong Gao, Jia Li, Fei Li

**Affiliations:** 1 Department of General Surgery, Xuanwu Hospital, Capital Medical University, No. 45, Xicheng, Beijing, 100053 People’s Republic of China; 2 Clinical Center for Acute Pancreatitis, Capital Medical University, No. 45, Xicheng, Beijing, 100053 People’s Republic of China

**Keywords:** Walled-off necrosis, Laparoscopic surgery, Complications, Sinistral portal hypertension

## Abstract

**Background:**

Laparoscopic transgastric necrosectomy (LTGN) has been used in treatment of walled-off pancreatic necrosis (WON) for more than a decade. However, the safety and effectiveness of LTGN for WON with sinistral portal hypertension was still unclear.

**Methods:**

WON patients with sinistral portal hypertension treated in our department between January 2011 and December 2018 were included and retrospectively analyzed in this study. Patients were divided into two groups according to different surgical approaches, LTNG or laparoscopic assisted trans-lesser sac necrosectomy (LATLSN). Perioperative and long-term outcomes were compared between two groups.

**Results:**

312 cases diagnosed with WON were screened and 53 were finally included in this study. Of the included patients, 21 and 32 cases were received LTGN and LATLSN, respectively. LTGN was associated with significantly lower morbidity than LATLSN (19.0% vs 46.9%, p = 0.04) and similar severe complication (Clavien–Dindo ≥ III) rate (12.5% vs 19.0%, p = 0.70). LTGN did not increase the rate of postoperative hemorrhage (9.5% vs 6.3%, p = 1.00) and mortality (9.5% vs 9.4%, p = 1.00). After 39 (11–108) months follow-up, the recurrence rate of WON and long-term complications were also comparable between groups.

**Conclusion:**

From current data, LTGN was safe and effective in treatment of WON patients with sinistral portal hypertension in terms of short- and long-term outcomes.

## Introduction

Surgical or endoscopic step-up debridement has been recommended as the standard therapy for necrotizing pancreatitis in terms of reducing short- and long-term complications when comparing with open surgery [1–3]. In the recent randomized clinical trials, endoscopic approach has been confirmed with lower rate of pancreatic fistulas and length of hospital stay and these results might result in a shift to the endoscopic management as treatment preference [4–6]. However, Dutch pancreatitis study group reported the bleeding requiring intervention in endoscopic approach was common with an incidence of about 22% [6]. Gastric varices secondly to sinistral portal hypertension might be an important risk factor for this complication. Comparing with endoscopic treatment, laparoscopic necrosectomy has its advantage in dealing with gastric hemorrhage by using suture or hemoclips. In addition, previous studies confirmed that transluminal surgery was associated with lower rate of pancreatic cutaneous fistula [5, 7]. Therefore, laparoscopic transgastric necrosectomy (LTGN) was performed in our medical center in selected cases since 2011. The effectiveness, safety, short-term and long-term outcomes of this approach for walled-off pancreatic necrosis (WON) with sinistral portal hypertension were focused in this study.

## Methods

### Patients

All medical records with diagnosis of acute pancreatitis between Jan. 2011 and Dec. 2018 were obtained from a prospectively maintained database. WON cases received surgical intervention were further screened. As necrosis confined to the lesser sac was the ideal indication for transgastric necrosectomy, then we carefully re-checked the imaging of computed tomography or magnetic resonance. Patients with necrosis confined to lesser sac and sinistral portal hypertension were finally included in this study. The diagnostic criteria of sinistral portal hypertension were as follows: ① superior mesenteric/splenic vein thrombosis; ② gastric or esophageal varices and ③ enlarged spleen on CT scan. The sinistral portal hypertension would be considered as any one of the above three signs was present in the patients with acute pancreatitis. Endoscopy was not performed routinely since CT scan was effective in identifying gastric or esophageal varices and had good patient acceptance [8, 9]. Two approaches, LTGN and laparoscopic assisted trans-lesser sac necrosectomy (LATLSN) were used in treatment of these patients. LTGN was preferred unless there were contraindications for laparoscopic surgery. Overall, infected complications developed in 40 to 70% of WON patients. In our center, the evidence of infected WON diagnosis was divided into two levels. Level I: presence of "bubble" within necrotic collections on contrast-enhanced CT; level II: clinical symptoms or general condition deterioration despite best support with laboratory infection index (blood leukocyte, C-reactive protein or serum procalcitonin) increased, and extrapancreatic infection was excluded. Fine needle aspiration was not routinely used. Antibiotics were used once infected pancreatic necrosis was suspected. Carbapenems or third-generation cephalosporin was empirically used until the results of bacterial culture and drug sensitivity test were obtained.

This study was approved by the Ethics Committee of Xuanwu hospital, Capital Medical University. Written informed consents were obtained from all patients or their legal representatives. All authors vouched for the accuracy and completeness of the data and analyses.

### Procedures

#### Laparoscopic transgastric necrosectomy (LTGN)

**Fig. 1:**
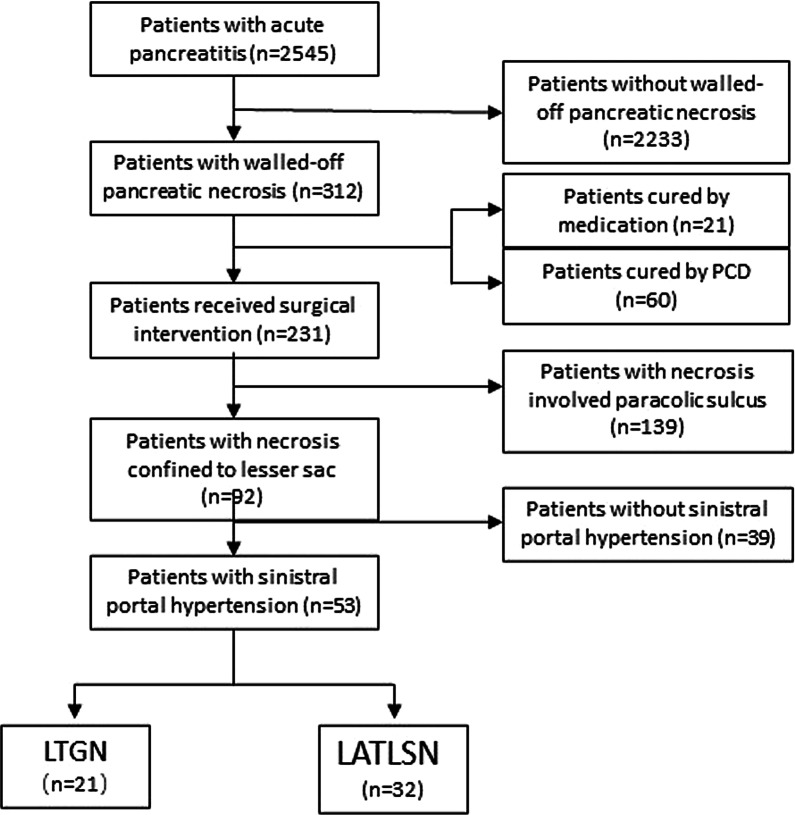
Representative preoperative CT imaging and intraoperative photos of laparoscopic trans gastric necrosectomy. Representative preoperative CT imaging revealed the walled-off pancreatic necrosis (WON) confined to lesser sac, gastric varices (black arrow) and enlarged spleen (red arrow) (**a**–**c**). Laparoscopic exploration showed that WON was located in the lesser sac (**d**). Opening the anterior wall of stomach (**e**). Laparoscopic ultrasound (LUS) was used to confirm the site, extent, and composition of WON (**f**). Opening the posterior wall of stomach (**g**). Laparoscopic staple was used to create gastrostomy in posterior gastric wall (**h**). Necrosectomy through gastrostomy (**i**). CT imaging after interventional treatment (**j**–**l**)

The LTGN procedure has been reported previously [10–12]. Usually, three or four ports method was used and placed similar to other foregut surgery. A 10 mm port was placed at the umbilicus to drive the camera. Another 12 mm port was placed in the right lower abdominal position to accommodate ultrasound probe and stapling devices. One or two 5 mm ports were placed at epigastric sites to facilitate the operation. After entering the abdominal cavity, adhesion was firstly released. Then, laparoscopic ultrasound (LUS) was used to confirm the site, extent, and composition of infection. Anterior wall of stomach was opened between stay sutures using ultrasonic scalpel. Again, using LUS by placing the ultrasound transducer directly into the posterior wall of the stomach to visualize the retrogastric collection was necessary to choose the site of posterior gastrostomy. Electrocautery and laparoscopic staple were used to create gastrostomy in posterior gastric wall, then the laparoscopy was placed into the necrosis cavity. Under the monitor of laparoscopy, necrosectomy was performed using blunt grasper. Hemorrhage from small vessels around the pancreas can be stopped by using hemoclips. Vigorous irrigation of the necrosis cavity was then performed to dislodge the small particulate solid matter. All the removed necrosis tissue was placed in the stomach. Another one or two laparoscopic staples were used close the anterior gastric wall. Representative intraoperative imagings were presented in Fig. 1. For biliary pancreatitis, cholecystectomy was performed simultaneous in selected cases.

#### Laparoscopic assisted trans-lesser sac necrosectomy (LATLSN)

The details of LATLSN has been described in our previous reports [13, 14]. Briefly, a 5–8 cm upper midline incision was made firstly. After entering the abdominal cavity, the gastrocolic ligament was separated under the gastric omental vascular arch. Gastrocolic ligament and the parietal peritoneum were circumferential sutured to establish the pathway and protect the abdominal cavity from pus. Then, mature necrotic tissue was removed by sponge forceps under the assistance of laparoscopy. Three or more 30–36 Fr drainage tubes were placed to reduce the risk of obstruction.

Minimally invasive debridement was technically demanding approaches. In our center, this procedure was performed by two senior surgeons who had experienced more than 30 cases of mini-invasive surgery at the beginning of this study.

### Postoperative management

Enhanced recovery programme after surgery was applied in our center. Antibiotics were routinely used and adjusted according to the results of bacterial culture. Vital signs were monitored every day. Characteristics and volume of the drainage fluid were also observed in patients with external drainage tubes. Low molecular weight heparin was routinely used for preventing deep vein thrombosis and progression of superior mesenteric/splenic vein thrombosis. With the help of nursing staff, patients were encouraged to ambulate on postoperative day (POD) 1. Oral feeding or enteral nutrition were restored on POD 2 unless gastric outlet or duodenum obstruction persisted or intolerance. CT reexamination was performed on POD 7 unless sepsis persisted or intraperitoneal hemorrhage occurred. In LATLSN patients, once the abscess was drained completely, amylase of drainage was examined. Somatostatin was used in patients with pancreatic fistula. Usually, the drainage tubes were withdrawn gradually on POD 14-21.

### Statistics

All continuous data were expressed as means ± SD or median with range and analyzed by Student *t* test. Categorized variables were compared using chi-square test or the Fisher exact test. All analyses were performed by SPSS 22.0 (SPSS Company, Chicago, IL, USA). *P* value < 0.05 was considered statistically significant.

## Results

### Patients

**Fig. 2:**
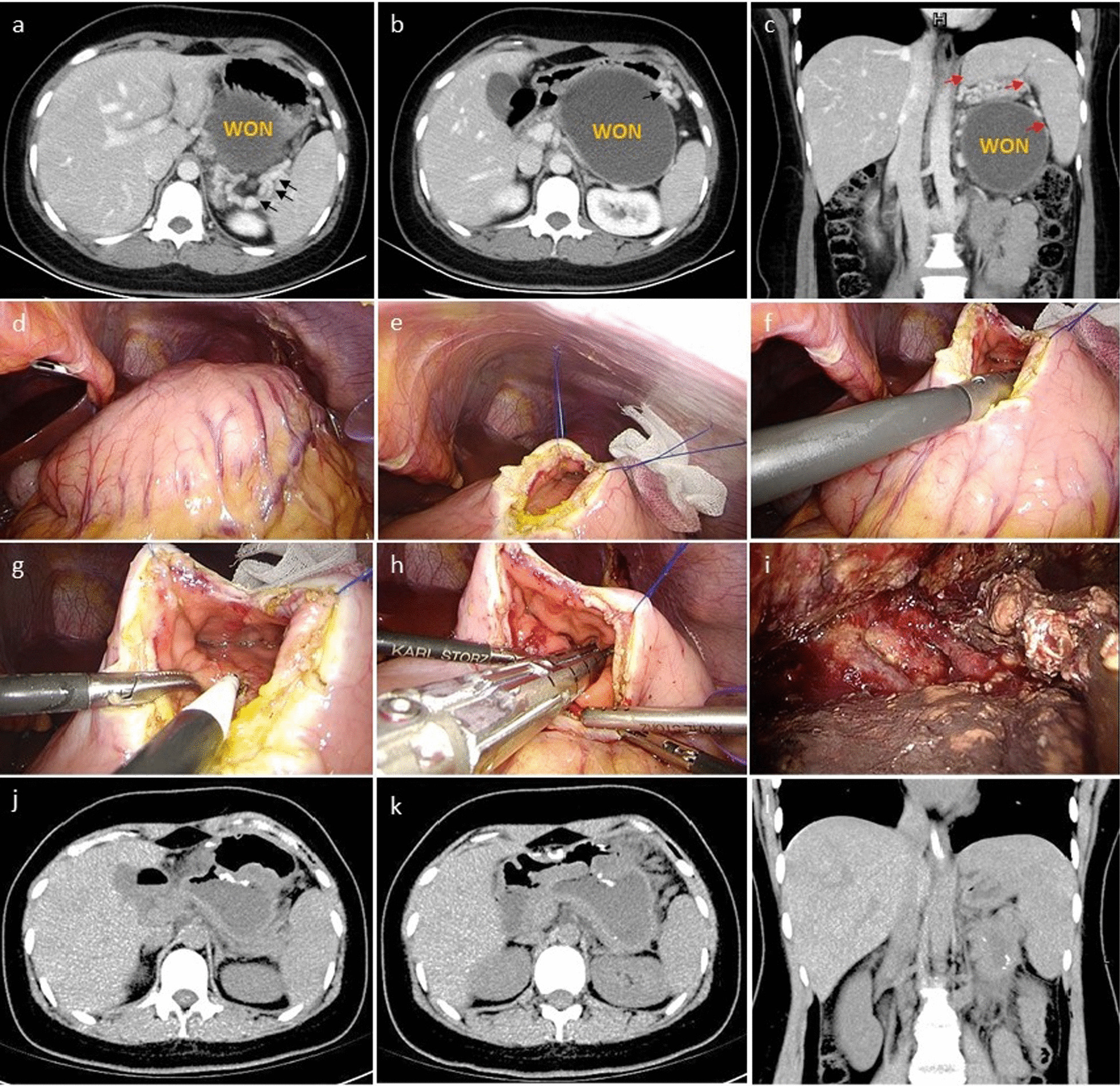
Flow chart of the included patients

From Jan. 2011 to Dec. 2018, 2545 patients with acute pancreatitis were treated in our hospital. Of them, 312 cases were diagnosed with WON. All of the patients underwent computed tomography as well as 79 patients underwent magnetic resonance. Peripancreatic infection occurred in 271 patients. All these patients were in accordance with level II evidence. However, only 39 cases (14.4%) had "bubble" sign (level I evidence). Surgical intervention was performed in 231 patients. Fifty-three patients with necrosis confined to lesser sac and sinistral portal hypertension were included in this study (Fig. 2). Of the included patients, 21 and 32 cases were received LTGN and LATLSN, respectively.

Among all the included patients, 29 were male and 24 were female. The median age was 56 (20–84). Cholelithiasis (43.4%) was the most common etiology of acute pancreatitis, followed by hypertriglyceridemia (20.8%) and alcohol abuse (13.2%). 43 cases were referred from other hospitals, and the median referral time was 28 (1–180) days. 21 cases experienced at least one positive blood culture and *E. coli* (33.3%, 11/33) was the most common pathogen, followed by *P. aeruginosa* (12.1%, 4/33), *A.baumanii* (9.1%, 3/33), *E.faecium* (6.1%, 2/33) and *K. pneumoniae* (3.0%, 1/33).

Patient characteristics of the 53 patients in LTGN and LATLSN groups were similar (Table 1).

**Table 1:** Patients and disease characteristics

	LTGN (n = 21)	LATLSN (n = 32)	P value
Age (years)	56 (21–84)	57 (20–79)	0.63
Male, n	11	18	0.78
Etiology of pancreatitis, n			0.08
Gallstones	8	15	
Hypertriglyceridemia	4	7	
Alcohol abuse	3	4	
Idiopathic and others	6	6	
Organ dysfunction, n			0.84
Renal	4	7	
Lung	3	6	
Cardiovascular	3	3	
Multiple	2	3	
ASA scores, n			0.96
III	11	17	
IV	10	15	
Charlson index of comorbidities	2 (0–7)	2 (0–8)	0.87
APACHE II socre, n			0.79
0–8	3	3	
8–16	14	24	
> 16	4	5	
CT severe index, n			0.92
0–6	5	8	
7–10	16	24	
Pancreatic necrosis, n			0.70
< 30%	2	2	
30–50%	6	6	
> 50%	13	23	
Inflammatory markers on operation time			
White cell count (× 10^9^/L)	12.3 (2.9–32.6)	13.2 (4.8–27.5)	0.62
C-reactive protein (mg/L)	156 (17.9–348)	148 (6.97–412)	0.30
Procalcitonin (ng/ml)	1.02 (0.07–100)	0.95 (0.09–74.8)	0.28
Maximum length of necrotic collection (cm)	9.3 (5.0–18.2)	8.6 (6.1–15.5)	0.39
Infected necrosis, n	19	29	1.00
Tertiary referral, n	16	25	0.87
Time to intervention from onset of pancreatitis (days)	37 (25–119)	35 (29–372)	0.69
Site of thrombus, n			0.59
Splenic vein	14	19	
Portal-splenic vein	7	13	

### WON characteristics

The maximum length of necrotic collection was 9.0 ± 3.1 cm according to the CT image. There were 5, 12, 40 patients had the pancreatic necrosis < 30%, 30%-50%, and > 50%, respectively. The CT severe index (CTSI) was 9.1 ± 2.0. Infection was confirmed in 48 case by positive culture from pancreatic necrosis obtained intraoperatively. *E. coli* (33.3%, 16/48) was the most common pathogen, followed by *P. aeruginosa* (29.2%, 14/48), *K. pneumoniae* (20.8%, 10/48) and *A. baumanii* (12.5%, 6/48). WON characteristics in the two groups were similar (Table 1).

### Patients outcomes [1]

Overall, 48 patients were successfully treated by laparoscopic surgery, and 5 cases died because of uncontrol sepsis or severe hemorrhage. The overall mortality was 9.4%. Postoperative complications were occurred in 19 patients with overall morbidity 39.6%. LATLSN was associated with significantly higher morbidity than LTGN (46.9% vs 19.0%, p = 0.04). However, the severe complication (Clavien–Dindo ≥ III) rate was similar in 2 groups (12.5% vs 19.0%, p = 0.70). Pancreatic fistula occurred 12 (37.5%) patients and was the most common short-term complication in LATLSN group. Of these patients, 10 recovered after somatostatin treatment, and the other 2 cases received ERCP with pancreatic duct stenting. Hemorrhage was occurred in 2 (9.5%) patients in LTGN group, which was similar with LATLSN group (6.3%, p = 1.00). Two patients succeeded in hemostasis by DSA combined with coil embolization, and others died (Table 2).

**Table 2:** Patients outcomes

	LTGN (n = 21)	LATLSN (n = 32)	P value
Mortality, n	2	3	1.00
Postoperative morbidity, n	6^a^	17^b^	0.04
Hemorrhage	2	3	
Pancreatic fistula	0	12	
Intra-abdominal infection	3	3	
Enterocutaneous fistula	1	1	
Clavien–Dindo ≥ III complications, n	4	4	0.70
Additional percutaneous drainage, n	2	2	1.00
Additional surgery, n	3	5	1.00
ICU stay (days)	6 (2–32)	5 (1–21)	0.76
Postoperative hospital stay (days)	13 (6–43)	11 (6–38)	0.45
Recurrent WON, n	2	2	1.00
Repeated surgery, n	1	1	1.00
Long-term complications, n	5	10	0.56
New-onset diabetes	3	3	
Pancreatic exocrine insufficiency	2	3	
Incisional hernia	0	0	

^a^Two patients in LTGN group had multiple complications, one with hemorrhage and colonic fistula, and another with hemorrhage and intra-abdominal infection

^b^Two patients were complicated with hemorrhage and pancreatic fistula

After initial surgery, infected symptoms in 12 patients were uncontrolled. Four and 8 patients received additional percutaneous drainage and surgery, respectively. Three patients died after second or third operation because of multiple organ dysfunction resulting from uncontrol sepsis shock. The mortality was similar in 2 groups (9.5% vs 9.4%, p = 1.00).

After 39 (11–108) months follow-up, 4 patients underwent WON recurred, and all recurrence occurred within 12 months. The method of surgery was not associated with recurrence. Two patients received repeated surgery, and were successfully treated after single operation. Long-term complications were occurred in 15 (28.3%) patients with new-onset diabetes in 6 cases, followed by pancreatic exocrine insufficiency (5, 9.4%) and incisional hernia (4, 7.5%). All case with incisional hernia underwent surgical repair and one received laparoscopic cholecystectomy for gallstone, simultaneously. The long-term outcomes were comparable between two groups (Table 2).

## Discussion

Secondly infection was a severe late complication and common cause of death after necrotizing pancreatitis. In this study, 48 (90.1%) patients were confirmed with infection by positive culture from pancreatic necrosis, which was higher than previous studies [3, 5]. This might be resulted from the high rate of tertiary referral (81.1%, 43/53) and most of the patients had been diagnosed with infection in previous hospitals. Timing of definitive surgery was an important factor affecting the outcome of infected pancreatic necrosis (IPN) patients. Lessons from open necrosectomy demonstrated early operation was associated with extremely high complication rate and mortality [15, 16]. Therefore, delayed surgery was recommended by current guidelines [17, 18]. In our clinical practices, we tried our best to postpone the operation to 4 weeks after the onset of the disease. However, we believed percutaneous drainage should be performed immediately once infection was considered.

This paper focus on the WON patients with sinistral portal hypertension, the varicose veins puts forward special requirements for the implementation of pancreatic necrotic tissue removal. In fact, splanchnic (SVT) or portosplenomesenteric (PSVT) venous thrombosis was not rare complication after acute pancreatitis with the incidence of 16.6–25.5%, which might result in sinistral portal hypertension [19, 20]. Previous studies have clearly demonstrated the risk factors, including red blood cell specific volume (HCT), D-dimer, serum amylase, APACHE-II score, and Ranson sore, for SVT or PSVT [21, 22]. About one fourth of the patients developed variable symptomatic manifestations including gastrointestinal bleeding, persistent ascites, oral intake intolerance and even hepatic infarction [23, 24]. In addition to SVT or PSVT, other venous thromboembolism (VTE) in necrotizing pancreatitis, including extremity deep venous thrombosis and pulmonary embolism was also common with the incidence of 16% and 6%, respectively [25]. Previous study showed male gender, history of previous deep venous thrombosis, infected necrosis, development of organ failure, and development of respiratory failure were identified as risk factors for VTE [25]. Recent systemic review demonstrated that about 46.5% patients received anticoagulation therapy, However, rates of recanalization of veins in the treated and non-treated groups were comparable [26]. In our center, anticoagulation therapy was routinely used since we believed it was important to prevent deep venous thrombosis and fatal pulmonary embolism. We did not find the increased rate of bleeding complications.

The choice of surgical method depends on the specific situation of the patient, and the occurrence of serious complications (such as bleeding during surgery) should be minimized. Surgical or endoscopic mini-invasive debridement has been widely performed in treatment of IPN with promising results, which was recommended by many guidelines. Patients with necrosis confined to lesser sac were special since different approaches, including laparoscopic transgastric, trans-lesser sac, endoscopic transluminal and trans-retroperitoneal approach can be used. In our center, endoscopic transluminal surgery was not routinely performed. The reasons were as follows [13]. First, endoscopic drainage and debridement were still technique demanding. Only one gastroenterologist in our hospital can performed this procedure expertly. It might be not available for IPN patients when necessary. On the other hand, percutaneous drainage and laparoscopic surgery was in the hands of the surgeons and can be performed at any time. Second, endoscopic therapy was much more expensive than surgical therapy in China. Many patients cannot afford the cost of endoscopic treatment, especially when multiple procedures were needed. Third, the most important reason for these patients, was the concerns about the bleeding complications especially in patients with sinistral portal hypertension. In this study, the overall hemorrhage rate was 9.4%, and none of the patients developed intraoperative bleeding from gastric varices in LTGN group, which means LTGN was safe in treatment of WON with sinistral portal hypertension. Postoperative hemorrhage was potential lethal complication after debridement surgery. Three cases (5.7%) died of severe hemorrhage from splenic artery. For the early postoperative bleeding, immediately surgery or arteriography should be performed to identify the criminal vessels. Previous studies confirmed that the common bleeding site after IPN included branches of splenic artery, superior mesenteric artery, left gastric artery, gastroduodenal artery and left colonic artery. Bleeding from the branches of the splenic and left gastric arteries can be embolized safely without serious consequences. In our center, there were cases of colonic leakage after left colonic artery embolization and duodenal fistula after gastroduodenal artery embolization. In case of emergency massive hemorrhage, all drainage tubes should be removed at the bedside, and packing hemostasis performed immediately, then transferred the patient to the intervention center or operating room.

Compared with LATLSN, LTGN has some advantages in reducing the incidence of short- and long-term complications. Pancreatic fistula was common in LATLSN group with incidence of 37.5%, which was significantly higher than LTGN group. In studies comparing surgical with endoscopic approach, the rate of pancreatic fistula in surgical group was similar with our report [5, 6]. Pancreatic fistula will prolong the duration of intubation and hospital stay [27]. Additional endoscopic or surgical therapy might be required in some patients. In our center, patients with pancreatic fistula were routinely given somatostatin. And 10 out of 12 cases in this study recovered in 12 weeks without any additional intervention. The other 2 cases received endoscopic stents treatment and were recurred in 4 weeks. In LTGN group, 76.2% of the patients were covered after single operation, only 2 (9.5%) and 3 (14.3%) patients needed additional percutaneous drainage and endoscopic surgery, respectively, which was comparable with the results of endoscopic treatment [6]. Incisional hernia was a common long-term complication after LATLSN surgery with the incidence of 12.5%. However, LTGN could avoid this complication completely. Furthermore, LTGN did not increase the rate of WON recurrence and other long-term complications, including new-onset diabetes and pancreatic exocrine insufficiency. Therefore, LTGN combined the advantages of less complications in endoscopic surgery and high efficiency in surgical approach [11, 27]. Even in WON patients with sinistral portal hypertension, LTGN did not increase the risk of hemorrhage.

This was a retrospective study with a relatively small sample size, which may lead to a certain recall and case-selected bias. As the laparoscopic or open surgery were still the first choice to deal with WON in China, this study did not include the cases received the endoscopic debridement. However, we believed this initial experience about laparoscopic transgastric approach for WON with sinistral portal hypertension provided more therapeutic selection in dealing with these patients.

## Conclusion

From current data, LTGN was safe and effective in treatment of WON patients with portosplenomesenteric venous thrombosis and sinistral portal hypertension in terms of short- and long-term outcomes.

## Data Availability

The data that support the findings of this study are available from the corresponding author upon reasonable request. Emails could be sent to the address below to obtain the shared data: feili36@ccmu.edu.cn.
